# IL-4 enhances survival of in vitro-differentiated mouse basophils through transcription-independent signaling downstream of PI3K

**DOI:** 10.1038/s41419-018-0754-z

**Published:** 2018-06-18

**Authors:** Ramona Reinhart, Thomas Kaufmann

**Affiliations:** Institute of Pharmacology, University of Bern, Bern, Switzerland

## Abstract

Interleukin 4 (IL-4) is a critical cytokine implicated with T_H_2 immune reactions, which are linked to pathologic conditions of allergic diseases. In that context, the initiation of T_H_2 responses can critically depend on early basophil-derived IL-4 to activate T-cell responses, which then amplify IL-4 secretion. As a pleiotropic cytokine, IL-4 acts on a broad variety of hematopoietic and non-hematopoietic cells. However, the effect of IL-4 on basophils themselves, which are emerging as relevant players in allergic as well as autoimmune diseases, was only scarcely addressed so far. Here we used in vitro-differentiated mouse basophils to investigate the direct effects of IL-4 on cellular viability and surface expression of the high-affinity receptor for IgE, FcεRI. We observed that IL-4 elicits pronounced pro-survival signaling in basophils, delaying spontaneous apoptosis in vitro to a degree comparable to the known pro-survival effects of IL-3. Our data indicate that IL-4-mediated survival depends on PI3K/AKT signaling and—in contrast to IL-3—seems to be largely independent of transcriptional changes but effectuated by post-translational mechanisms affecting BCL-2 family members among others. Additionally, we found that IL-4 signaling has a stabilizing effect on the surface expression levels of the critical basophil activation receptor FcεRI. In summary, our findings indicate an important regulatory role of IL-4 on in vitro-differentiated mouse basophils enhancing their survival and stabilizing FcεRI receptor expression through PI3K-dependent signaling. A better understanding of the regulation of basophil survival will help to define promising targets and consequently treatment strategies in basophil-driven diseases.

## Introduction

The source of interleukin (IL)-4 in vivo is thought to derive upon activation from at least three different cell types, including mast cells, basophils as well as a subpopulation of T cells. Once released, IL-4 acts as a prominent cytokine in type 2 immune reactions fulfilling diverse functions. In T cells, upon activation of naive peripheral CD4^+^ T cells autocrine IL-4 drives their cellular growth and differentiation^1^. Consequently, naive T cells mature into T_H_2 cells leading to the initiation of T_H_2 immune reactions. In general, IL-4 represents a pleiotropic cytokine acting on different cells. Besides its substantial effect on the viability of T and B lymphocytes^2^, IL-4 is also implicated with tissue adhesion and inflammation leading to the recruitment of T cells and eosinophils (reviewed in ref. ^3^). Moreover, IL-4 promotes class switching in B cells for de novo synthesis of immunoglobulins, in particular IgE, which together with T_H_2 lymphocytes execute a protective host defense against parasite infections. However, allergen-specific T_H_2 reactions are also associated with atopic disorders and are recognized to take part in the pathogenic conditions of progressive systemic sclerosis, cryptogenic fibrosing alveolitis^4^, and in some forms of systemic autoimmune diseases^5^. Especially upon allergen crosslinking of the high-affinity IgE receptor, FcεRI, or through IgE-independent activation, de novo-synthesized cytokines such as IL-4 are released from mast cells and basophils^6^. Besides secreting IL-4, mast cells also directly respond to this cytokine. IL-4 serves not only as a growth factor for human intestinal mast cells but also enhances IgE-dependent mediator release^6^ and promotes de novo expression of other cytokines, such as IL-3, IL-5 and IL-13, whereas the production of IL-6 is suppressed^7^. Likewise, human intestinal mast cells were recognized to prolong their survival through IL-4-induced priming. By a reversible process, IL-4 leads to upregulation of mast cell proliferation as well as increased FcεRI expression^8,9^. Yet, IL-4 alone is not able to affect mast cell survival but strongly enhances mast cell proliferation and T_H_2-type cytokine production in presence of stem cell factor^8^. In terms of survival regulation, IL-4 was reported to induce the anti-apoptotic BCL-2 family members BCL-2 and BCL-X_L_ and increase survival of cultured bone marrow-derived mouse mast cells in a STAT6-dependent manner^10^. IL-4 was further seen to prevent cell death in multiple hematopoietic cell types through the activation of the PI3K/AKT pathway^2^. From studies with the IL-3-dependent myeloid progenitor cell line FDCP-2, it became clear that the effect of IL-4 is distinct from that of IL-3, activating specific non-redundant tyrosine phosphorylations strongly associated with PI3K signaling, while IL-3 was found to trigger PI3K activation only weakly^11^. With regards to the related eosinophils and neutrophils, conflicting effects of IL-4 on human eosinophils were reported^12,13^, whereas in human neutrophils, IL-4 was found to enhance general RNA synthesis, resulting in enhanced survival and activation of cytoskeletal rearrangements^14^.

Interestingly, basophils were recognized to release a considerable amount of IL-4 upon activation, which then serves as a critical source of early IL-4 to initiate T_H_2 immune reactions through primary T-cell activation^15^. Moreover, many physiological and pathological conditions were revealed to be linked to specific basophil-derived IL-4, which impacts on hematopoietic (T cells, B cells, ILC2s, and macrophages) as well as on non-hematopoietic cells (fibroblasts and endothelial cells) (reviewed in ref. ^16^). These include the protective effects against parasites and infectious bacteria but also certain allergic and autoimmune diseases. Moreover, several studies indicate that IL-4 increases histamine release of human basophils as well as mast cells^17,18^.

Although IL-4 potently affects multiple cell types within the hematopoietic system, its specific effect on basophils is poorly understood. In particular, it is not clear whether and how IL-4 directly influences basophil survival. In regard of the emerging role of basophils especially in T_H_2-related diseases, such as diverse allergic disorders^19^, mechanistic understanding of the regulation of basophil survival may offer novel insights into basophil mediated disease progression. To address this question, we took advantage of an established model of in vitro-differentiated mouse basophils, termed IL-3^cond^Hoxb8 basophils^20,21^, to investigate molecular mechanisms regulating mouse basophil survival.

## Results

### IL-4 induces dose-dependent basophil survival and stabilizes cellular granularity and size

**Fig. 1:**
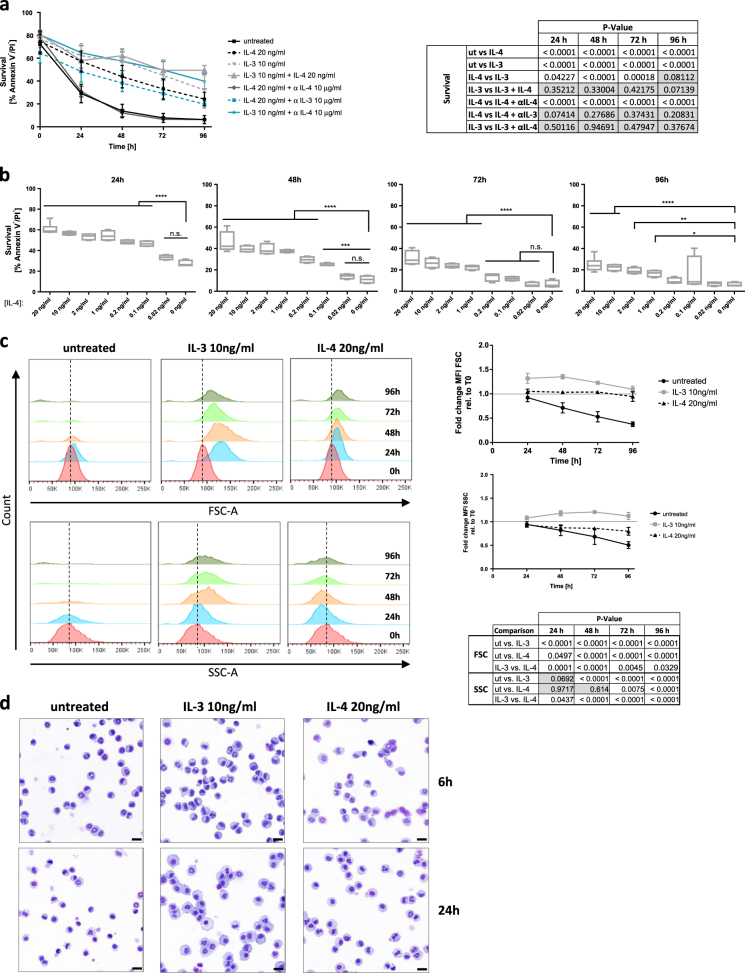
Dose-dependent increase of basophil survival by IL-4. **a** Survival of in vitro-differentiated basophils in presence or absence of recombinant cytokines (10 ng/ml IL-3, 20 ng/ml IL-4) or blocking antibody (anti-IL-4 and anti-IL-3, 10 μg/ml each; both antibodies are of the same isotype) over time was measured by flow cytometry using annexin V/PI exclusion (*n* ≥ 3, ±SD). Statistical analysis using multiple *t* test with Holm-Sidak post hoc for multiple comparison shows *p*-values of indicated comparisons, non-significant results highlighted in gray. **b** Quantification of GFP-annexin V/PI double-negative cells by flow cytometry illustrating both dose and time-dependent survival of in vitro-differentiated basophils upon IL-4 stimulation (24 h, 48 h, 72 h, and 96 h) (*n* ≥ 4, ±SD, two-way ANOVA with Dunnett’s multiple comparison correction, *α* = 0.05. *p < 0.05, **p < 0.01, ***p < 0.001, ****p < 0.0001). **c** Granularity and cellular size in untreated or cytokine-treated basophils was determined by side scatter (SSC) and forward scatter (FSC), respectively (*n* ≥ 3, ± SD). Table shows statistical analysis of quantified SSC and FSC graphs. *p*-values calculated by two-way ANOVA with Turkey’s multiple comparison correction. *p* < 0.05 is considered a statistically significant difference between the treatments. **d** Representative cytospins of in vitro-differentiated basophils upon 6 or 24 h of incubation without or with cytokine at indicated concentrations. Images were acquired with a Panoramic Midi slide scanner and analyzed by CaseViewer software, both from 3DHISTECH (Budapest, Hungary), scale bars correspond to 20 μm

Throughout this study, we traced the impact of IL-4 on survival of basophils using the IL-3^cond^Hoxb8 model of in vitro-differentiated mouse basophils^20,21^. Upon complete basophil maturation, in vitro-differentiated mouse basophils spontaneously underwent apoptotic cell death, as assessed by flow cytometric analysis of cellular viability over time (Fig. 1a). Interestingly, this effect could be significantly delayed by the administration of recombinant IL-4, even though the impact was less potent compared to IL-3, which is a well-known and critical pro-survival cytokine for basophils (Fig. 1a and ref. ^22^). Flow cytometric analysis confirmed the surface expression of CD124, the alpha subunit of the IL-4 receptor (Supplementary Figure S1). IL-4 did not further enhance IL-3 mediated basophil survival, indicating that the potent effect of IL-3 might have already reached its plateau. To prove the specificity for IL-4-induced survival, we administered neutralizing anti-IL-4 antibody, which completely abolished the IL-4-mediated, but not the IL-3-mediated, increase in survival (Fig. 1a). Furthermore, the addition of a neutralizing anti-IL-3 antibody did not reduce the IL-4 mediated effects, indicating that IL-4 does not enhance survival via autocrine IL-3 (Fig. 1a). As shown in Fig. 1b, IL-4 elicited both a dose- and time-dependent increase in basophil survival. Moreover, and similar to IL-3, addition of IL-4 prevented the loss of basophil granularity and decrease in cell size that occurs upon cytokine withdrawal, even though to lesser extents (Fig. 1c, d). Taken together, these data demonstrate that IL-4 triggers significant and IL-3-independent pro-survival signaling in in vitro-differentiated mouse basophils.

### IL-4-induced basophil survival is mediated through PI3K-dependent and largely transcription-independent signaling

**Fig. 2:**
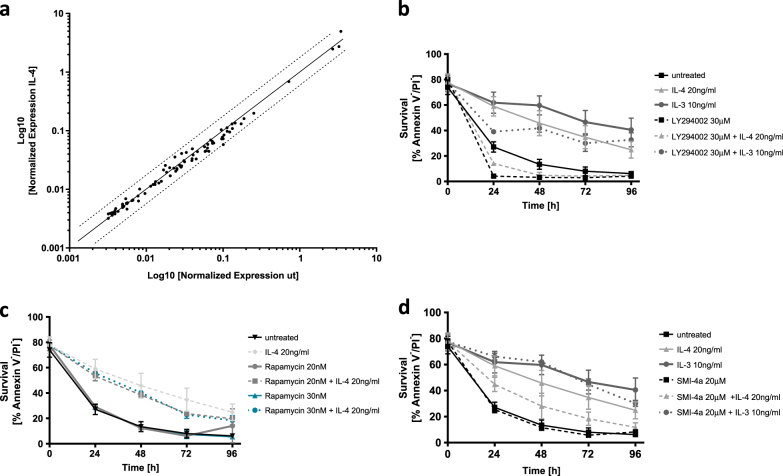
PI3K driven, but transcriptionally independent, regulation of IL-4-mediated basophil survival. **a** PCR array of apoptosis-related genes comparing mRNA levels of in vitro-differentiated basophils upon 3 h of incubation with or without 20 ng/ml IL-4. Using the SA Biosciences software, Ct values were normalized to the average of five different housekeeping genes (β actin, β2 microglobulin, Gapdh, Gusb, and Hsp90ab1) and linearized with the standard formula for qPCR (2^−(Ctsample−Ctcontrol)^). The *y*-axis corresponds to the expression of IL-4-treated cells, while the *x*-axis corresponds to the untreated controls. The continuous line depicts the equal expression level in both IL-4 and untreated basophils, whereas the dotted lines display a threshold of twofold increase or decrease of the mRNA expression comparing IL-4 over untreated. **b**–**d** Viability measurements of in vitro-differentiated basophils over time evaluated by GFP-annexin V/PI exclusion using flow cytometry (*n* ≥ 3, ± SD, statistics shown in Supplementary Table S2). **b** Using LY294002 at a concentration of 30 μM, the effect of PI3K inhibition was tested with or without cytokines (20 ng/ml IL-4 or 10 ng/ml IL-3). **c** The mTOR inhibitor rapamycin was added at two different concentrations with or without IL-4 co-treatment. **d** Administration of the selective PIM-1 inhibitor SMI-4a at a concentration of 20 μM alone or in combination with IL-4 or IL-3. Same data as in Fig. 1a are included in (**b**–**d**) for untreated, IL-4 or IL-3 treatments (*n* ≥ 5)

We recently reported that IL-3 mediates pro-survival signaling in basophils by transcriptional and post-translational activation of pro-survival BCL-2 family members^22^. Interestingly, and in contrast to IL-3, quantitative PCR array data showed that mRNA levels of apoptosis regulating genes only marginally changed upon IL-4 exposure (Fig. 2a). These results indicate that the pro-survival signaling of IL-4 is largely independent of gene transcription and may thus rather be mediated through post-transcriptional regulation. In hematopoietic cells, IL-4-mediated survival has been linked to PI3K > AKT signaling and was shown to result in specific and non-redundant tyrosine phosphorylation compared to IL-3, which triggers a weaker activation of PI3K in a factor-dependent myeloid cell line^2^. To test if the protective effect of IL-4 on basophil survival is indeed regulated by PI3K, we used the potent and stable PI3K inhibitor LY294002. As shown in Fig. 2b, the pro-survival effect of IL-4 could be completely abolished as soon as LY294002 was co-administered, while the protective effect of IL-3 was only partially diminished. Moreover, spontaneous cell death in the absence of specific cytokines was further enhanced by LY294002 implicating the relevance of PI3K for cell survival already at steady state (see also vehicle control in Supplementary Figure S2). Of note, the effect of IL-4-induced basophil survival as well as its dependence on PI3K could be confirmed in bone marrow-derived c-kit^−^CD49b^+^FcεRI^+^/IgE^+^ primary mouse basophils (Supplementary Figure S3a, b). Even though PI3K can activate the mTOR pathway, which is well known to regulate cell growth, proliferation, and survival^23^, inhibition of mTOR by rapamycin had no significant effect on basophil viability suggesting that mTOR is not critical for IL-4 mediated survival in that context (Fig. 2c).

Given that Provirus integration site for Moloney leukemia virus 1 (PIM-1) was reported to be an important kinase for human basophil survival downstream of IL-3, and that LY294002 at a concentration of 30 µM also inhibits PIM-1^21,24^, we tested the effect of a specific PIM-1 inhibitor, SMI-4a. As shown in Fig. 2d, treatment with SMI-4a partially but significantly counteracted IL-4-mediated survival (*p* = 0.0083/0.0172/0.0089/0.0058 at time points 24/48/72/96 h, multiple *t* test with post hoc of Holm-Sidak correction; see entire statistics of Fig. 2b–d in Supplementary Table S2). In summary, these data critically identify transcription-independent events downstream of PI3K to mediate survival of mouse basophils in response to IL-4, while PIM-1 may also partially contribute.

### IL-4 induces post-translational modifications through protein phosphorylation downstream of AKT

**Fig. 3:**
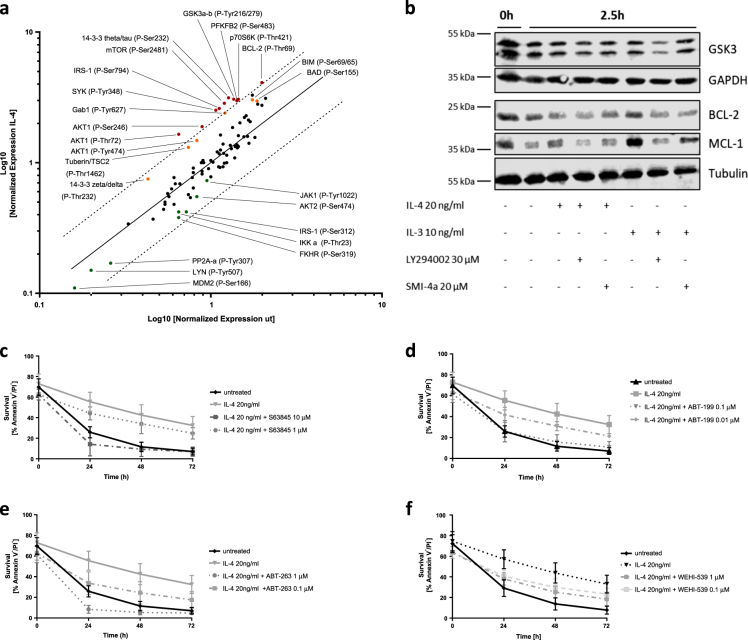
IL-4 triggers rapid changes in AKT-mediated protein phosphorylation pattern. **a** Data of a protein phospho-array directed against a distinct set of phosphorylation sites involved in the AKT-dependent signaling cascade comparing IL-4 treated in vitro-differentiated (*y*-axis) to untreated basophils (*x*-axis). The continuous line corresponds to equal phosphorylation status in IL-4 and untreated, whereas the dotted lines represent the arbitrary chosen threshold of a twofold increase or decrease of IL-4-treated over untreated after 2.5 h of incubation. See complete list and fold increase/decrease in phosphorylation in Supplementary Table S1. **b** Western blot of in vitro-differentiated mouse basophils after indicated time points upon cytokine treatment with IL-4 (20 ng/ml) or IL-3 (10 ng/ml) with or without kinase inhibitors (PI3K inhibitor LY294002, 30 μM; PIM-1 inhibitor SMI-4a, 20 μM), depicted as a representative example using near-infrared fluorescence (*n* ≥ 3). Flow cytometric analysis of in vitro-differentiated basophils upon (**c**) S63845, (**d**) ABT-199, (**e**) ABT-263 or (**f**) WEHI-539 administration at indicated concentrations with and without cytokines (*n* = 4, ±SD, see statistical analysis in Supplementary Table S3). Same data as in Fig. 1a are included in (**c**–**f**) for untreated or IL-4 treatments (*n* ≥ 5)

The above results point toward an important transcription-independent contribution of the PI3K > AKT signaling pathway downstream of IL-4 mediated basophil survival. In support of this, phospho-AKT protein levels increased in response to IL-4 treatment (Supplementary Figure S4a). A protein phosphorylation array integrating a broad range of phosphorylation targets of protein kinase B, PKB/AKT, was performed to compare the phosphorylation profiles of untreated vs. IL-4 treated basophils. This resulted in a list of potential candidates that may act as important downstream targets (Fig. 3a and see complete data list in Supplementary Table S1). Candidates included glycogen synthase kinase-3 (GSK-3), which is known to be inactivated through AKT-mediated phosphorylation, and which in its active state was shown to inactivate the pro-survival BCL-2 family member MCL-1 through phosphorylation at S159 upon IL-3 deprivation^25,26^. Therefore, the identified increase in GSK-3 phosphorylation upon IL-4 suggests its inhibition and subsequent stabilization of MCL-1 to enhance the survival of in vitro-differentiated basophils, which was indeed supported by a slight increase in MCL-1 protein levels after IL-4 addition compared to untreated controls (Fig. 3b and see quantification in Supplementary Figure S4b). Moreover, co-administering PIM-1 or PI3K inhibitor reversed IL-4 or IL-3 induced increase in MCL-1 (Fig. 3b). Nevertheless, using the novel and highly specific MCL-1 inhibitor S63845 at a concentration of 1 μM^27^, we could see only a minimal decrease in basophil viability. Only at a very high concentration of 10 μM S63845 was the pro-survival effect of IL-4 completely abolished (Fig. 3c).

Likewise, our results implicate a post-translational activation of BCL-2 by IL-4 (Fig. 3a), as the activating phosphorylation of BCL-2 at Thr-69 enhances its pro-survival function^28,29^. Supporting the importance of IL-4 mediated basophil survival, specific inhibition of BCL-2 by the BH3 mimetic ABT-199 totally overcame IL-4-induced survival already at concentrations as low as 0.1 μM (Fig. 3d). The simultaneous inhibition of BCL-2 and BCL-X_L_ by ABT-263 resulted in an even more potent cell death induction (Fig. 3e). Interestingly, and similar to IL-3 mediated mouse basophil survival, the inhibition of BCL-X_L_ alone by WEHI-539 was already sufficient to partially counteract the pro-survival effect of IL-4 (Fig. 3f) (see statistical analyses of Fig. 3c–f in Supplementary Table S3). Additionally, IL-4 exposure led to a reportedly inactivating hyperphosphorylation of the pro-apoptotic BH3 only proteins BAD and BIM (Fig. 3a)^30,31^. Along the same line, we found a potent increase in phosphorylation of 14-3-3 by IL-4, which was shown to result in its stronger binding and blocking of the pro-apoptotic functions of BIM and BAD^32,33^, which would support the enhanced survival of basophils upon IL-4 exposure.

Besides, we also observed an increase in phosphorylation of spleen tyrosine kinase SYK and de-phosphorylation of LYN (Fig. 3a), both of which are activating modifications^34,35^. LYN and SYK are in this respect interesting because they are known to function upstream of AKT and downstream of FcεRI, one of the most important activation receptors of basophils. However, it remains unclear if and how IL-4 receptor signaling crosstalks to FcεRI in basophils.

### IL-4 stabilizes the surface expression levels of FcεRI through a PI3K-dependent mechanism

**Fig. 4:**
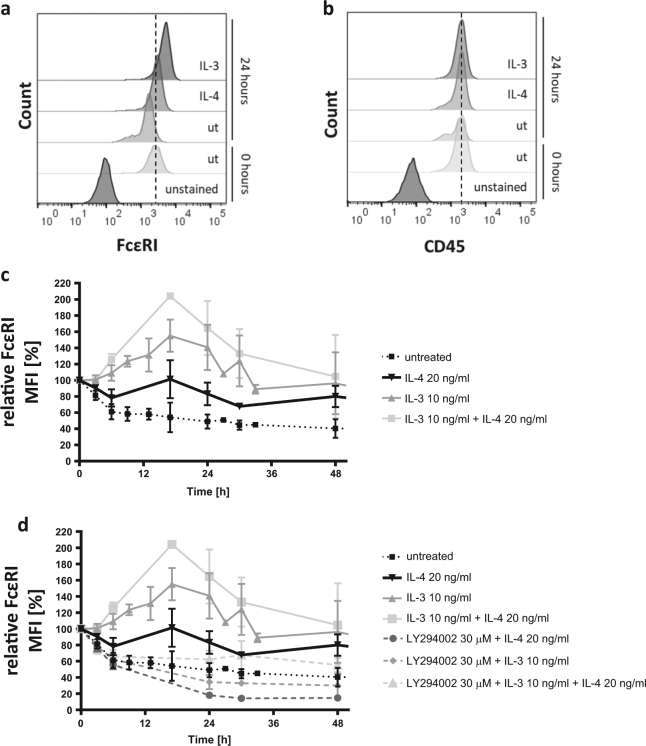
IL-4 stabilizes the surface expression level of FcεRI on in vitro-differentiated basophils in a PI3K-dependent manner. Flow cytometric analysis of FcεRI (**a**, **c**, **d**) and CD45 (**b**) surface expression levels, gated on viable cells and depicted as (**a**, **b**) representative histograms at 24 h of incubation and (**c**, **d**) of mean fluorescence intensity (MFI) relative to the 0 h time point, over time (*n* ≥ 3, ±SD, see statistical analysis of (**c**, **d**) in Supplementary Table S4). **c** Comparison of FcεRI expressions upon single or combinational cytokine administration at indicated concentrations compared to untreated over 48 h. **d** Combinational treatment with 30 μM of the PI3K inhibitor LY294002 with either IL-4 and/or IL-3. Same data from (**c**) are included

In human mast cells, IL-4 was shown to increase the surface expression of FcεRI^36,37^. Because LYN and SYK, both proximal kinases downstream of FcεRI, were identified in the protein phospho-array to be activated upon IL-4 stimulation (Fig. 3a), we hypothesized that IL-4 receptor signaling might also be linked to FcεRI expression in basophils. Therefore, we investigated the surface expression levels of FcεRI on in vitro*-*differentiated basophils upon IL-4 exposure over time. Already 24 h after cytokine exposure, we found increased FcεRI surface expression compared to its initial level (Fig. 4a), while the unrelated surface protein CD45 did not show such changes (Fig. 4b). As shown in Fig. 4c (see statistical analysis in Supplementary Table S4), surface FcεRI was significantly stabilized over time by IL-4 compared to control, yet, to lower extents compared with IL-3. Interestingly, however, we observed that the increase of FcεRI surface expression after IL-3 administration was further, and significantly, increased as well as prolonged upon co-administration of IL-4 (Fig. 4c). These data indicate that IL-4 contributes to an increase of surface FcεRI levels in basophils. Of note, similar prolongation of increased FcεRI expression could be obtained by the re-administration of IL-3 after 33 h of incubation (Supplementary Figure S5a). We further found a strong negative correlation between the expression levels of FcεRI and the CD123 unit of the IL-3 receptor, even though this may likely be caused by increased endocytic uptake of IL-3/CD123 complex as a form of negative feedback regulation (Supplementary Figure S5b, c). The cytokine-induced increase of surface FcεRI was totally abolished by the co-administration of the PI3K inhibitor LY294002 (Fig. 4d) for both, single or combined treatments with IL-4 and IL-3, respectively. These data indicate that both IL-4 and IL-3 positively regulate surface expression levels of FcεRI through PI3K signaling-dependent mechanisms. The stabilization of surface FcεRI expression by IL-4, and the role of PI3K therein, were confirmed in c-kit^-^CD49b^+^FcεRI^+^/IgE^+^ bone marrow-derived primary mouse basophils (Supplementary Figure S3c).

## Discussion

Mast cells, which share many features with basophils, are potently influenced by IL-4. Specifically, IL-4 was shown to serve as a growth factor and to enhance IgE-dependent degranulation of human intestinal mast cells^6^, to promote cytokine expression^3^ and to enhance human mast cell survival^38^. Here we could show that IL-4 likewise enhances viability of in vitro-differentiated mouse basophils in a dose- and time-dependent manner. Even though CD124 was strongly expressed on basophils and IL-4 enhanced basophil viability independently of IL-3, we cannot exclude the possibility that autocrine signaling of yet unknown factors downstream of IL-4 also contribute to the pro-survival effect. Although IL-3 constitutes the most potent survival factor for basophils, IL-4 could partially compensate for the absence of IL-3. Of note, Bailey et al.^39^ showed that the viability of mouse bone marrow-derived mast cells is negatively affected by long-term (>10 days) presence of IL-4 in the culture medium. But due to their much shorter lifespan, long-term effects of IL-4 on basophil viability could not be investigated here.

Although IL-4 is well known to induce transcription through the transcription factors JAK/STAT6^40^, we did not observe transcriptional changes of key apoptosis regulating genes at short time points after IL-4 stimulation. This suggests an important contribution of post-translational regulation of basophil survival by IL-4. We accordingly identified the PI3K pathway to play a major role in IL-4 mediated survival of basophils, whereas PIM-1 was found to partially contribute as well. The data from the AKT phospho-array strongly indicate the involvement of AKT downstream of PI3K; however further investigations are needed to assess if PI3K-dependent but AKT-independent pathways also contribute. As reported by others, growth factors and cytokines can enhance PIM-1 protein levels through transcriptional regulation that might operate in synergy with AKT to overcome pro-apoptotic signaling^41^. Even though former studies suggested the importance of PIM-1 in IL-3-mediated human basophil survival, using a specific PIM-1 inhibitor, we could not confirm this effect in in vitro-differentiated mouse basophils. Those results point toward possible differences in the regulation of basophil survival between humans and mice that need to be further investigated.

Although we saw a prominent activation of mTOR and its downstream substrate p70S6K in the phospho-array (Fig. 3a), inhibition of mTOR by rapamycin did not affect basophil viability. Those results indicate the dispensability or redundancy of mTOR signaling in the anti-apoptotic signaling of IL-4 in our cellular system. On the other hand, we observed an inactivating phosphorylation of GSK-3 in response to IL-4, which has been described to be mediated by AKT. Even though we did not detect a significant change in protein level (Fig. 3b), subsequent loss of GSK-3 activity may directly impact on protein stabilization, such as of the pro-survival protein MCL-1. This was supported by the increase in MCL-1 protein levels upon IL-4 administration, even though the effect was not as clear as seen with IL-3. We recently reported that MCL-1 is not the most critical pro-survival BCL-2 family member to mediate IL-3-driven survival of mouse basophils^22^. We obtained similar results in the context of IL-4 using the specific MCL-1 inhibitor S63845. In contrast, we previously identified MCL-1 as a critical pro-survival protein in human basophils^22^, again underlining important differences between mouse and human basophils.

Previous studies showed that phosphorylation of BCL-2 importantly regulates fate decision of IL-3 mediated survival and even suggested the necessity of BCL-2 phosphorylation for its full and potent survival phenotype^22,28,29,42^. Likewise, we found increased phosphorylation of BCL-2 in IL-4 mediated survival indicating the impact of the enhanced pro-survival function of BCL-2 in IL-4-induced basophil viability. This is supported by our data on BCL-2-, or BCL-2- and BCL-X_L_-selective BH3 mimetics (ABT-199 and ABT-263), respectively, both of which completely abrogated IL-4-mediated survival at low concentrations. However, by comparing the BCL-X_L_ specific inhibitor WEHI-539 to ABT-199, it became clear that BCL-2 might be of higher importance for IL-4-mediated basophil survival. On the other hand, we also found changes in AKT-mediated phosphorylation of pro-apoptotic proteins downstream of IL-4. As previously shown, survival factors such as IL-3 block the apoptotic activity of BAD through phosphorylation of specific serine residues^43–45^. Interestingly, it was shown that the BAD phosphorylation at Ser-155 requires the preceding phosphorylation at Ser-136 mediated by PI3K/AKT^45^. This leads to the recruitment of 14-3-3 proteins to increase the accessibility of Ser-155 to other survival-promoting kinases^44^. These findings support our results of increased BAD and 14-3-3 phosphorylations and suggest that these proteins may further contribute to IL-4-mediated basophil survival.

Of interest is the finding that IL-4 signaling crosstalks to the regulation of FcεRI surface expression in mouse basophils. Our data support the work by others, showing that IL-4 leads to the upregulation of FcεRI expression in human mast cells^8,9,36,37^. This effect might be of relevance, as FcεRI, upon antigen crosslinking, is among the most important activating receptors on basophils. Additionally, IL-4-mediated increase of surface FcεRI would further confirm the finding that IL-4 increases the reactivity of human basophils^17^. Nevertheless, whether the increase in FcεRI on mouse basophils provokes enhanced mediator release upon activation similar to mast cells still needs to be clarified. Interestingly, and in contrast to the effect on basophil viability, the combinational treatment of in vitro-differentiated basophils with IL-4 and IL-3 showed an additive effect on increase of surface FcεRI expression. This indicates that the regulation of FcεRI surface expression is regulated through different pathways. Particularly in basophils, SYK as well as LYN are of high importance in the initiation of FcεRI signaling, leading to histamine release and degranulation of basophils and mast cells^46^. The clustering of FcεRI on the surface of basophils (as well as mast cells) triggers a cascade of downstream signals including the activation of the Src family protein tyrosine kinase LYN and subsequent recruitment of SYK tyrosine kinase. Based on our findings, including the activation of LYN and SYK downstream of IL-4, the process of IL-4-induced basophil survival may be linked to the surface receptor regulation of FcεRI, and its downstream signaling.

Sharing the IL-4Rα chain with IL-4, IL-13 might likewise enhance basophil viability. However, as shown in Supplementary Figure S6a, IL-13 alone was not sufficient to critically prolong the lifespan of basophils. Yet, a slight increase was still measurable. This suggests, that during inflammatory responses, the mixture of distinct cytokines might contribute to enhancement of cell survival. Even the alarmin cytokine IL-33, which is known to play a pivotal role in allergic inflammation and directly affects human basophil function^47^, was found to partially augment basophil survival rate at earlier time points (24 and 48 h), but did not improve viability upon simultaneous stimulation with IL-3 (Supplementary Figure S6b). This indicates that—besides IL-3—basophil viability is influenced by various pro-inflammatory cytokines, which potentially impact basophil lifespan during inflammatory reactions.

**Fig. 5:**
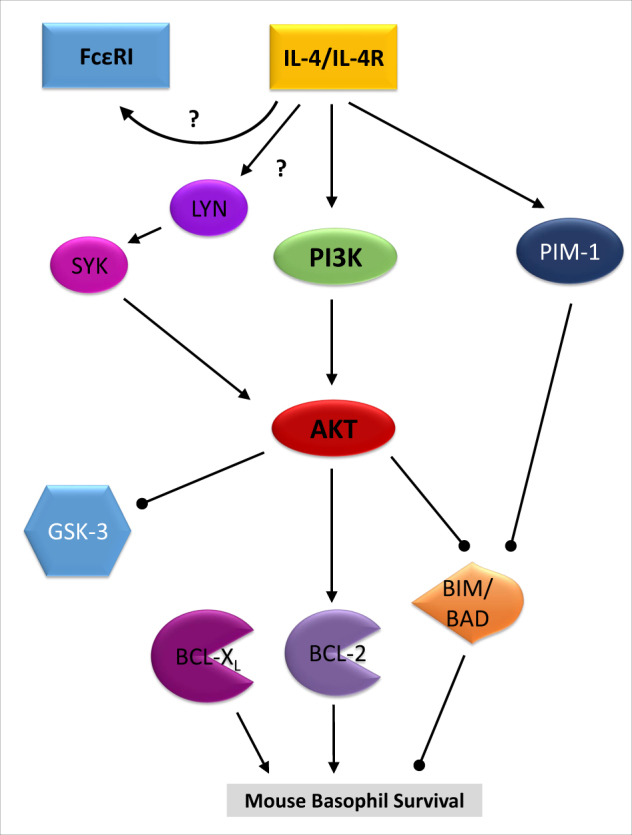
IL-4-mediated survival and surface FcεRI stabilization. Cartoon depicting a model of how IL-4 may lead to increased mouse basophil survival through the kinase activation of PI3K > AKT and PIM-1, which results in stabilization of pro-survival proteins like BCL-2 and destabilization of pro-apoptotic proteins such as BIM and BAD. Moreover, beside the stabilization of FcεRI surface expression through IL-4 signaling, IL-4 was also seen to affect SYK and LYN. However, the exact mechanism of SYK and LYN activation as well as the link between IL-4 signaling and FcεRI expression is not clear

In summary, we revealed the potential of IL-4 to enhance the viability of mouse basophils, which is critically mediated through PI3K > AKT signaling and involves important post-translational mechanisms (summarized in Fig. 5). However, not only the significant impact of IL-4 on basophil survival but also its positive effect on the surface expression level of FcεRI implicates the crucial importance of IL-4-mediated signaling on basophils and its specific action during immune responses (Fig. 5). In particular, during T_H_2 responses, which are associated with a potent release of IL-4 and consequently contribute to diverse diseases such as allergic and auto-inflammatory disorders, improved understanding of the regulation of basophil survival will help to better understand basophil-related pathogenesis and may offer novel treatment strategies.

## Materials and methods

### Mice and reagents

C57BL/6J mice were maintained under pathogen-free conditions in individually ventilated cages (IVC). Animal experiments were approved by the animal experimentation review board of the canton of Bern (BE12/14, BE138/16).

RPMI-1640 AQmedia™, propidium iodide (PI), 4-hydroxytamoxifen (4-OHT), and SMI-4a were purchased from Sigma-Aldrich Chemie GmbH (Buchs, CH). Fetal calf serum (FCS, Sera Pro, ultra-low endotoxin) was purchased from Pan Biotech (Aidenbach, DE), penicillin–streptomycin from Gibco (Thermo Fisher Scientific, Waltham, MA, US). As a source of mouse IL-3, WEHI-3B cell-conditioned medium was used and produced as previously described^21,48^. Recombinant mouse IL-4, IL-3, IL-33, and IL-13 were purchased from Peprotech (London, UK). Q-VD-OPh was purchased from SM Biochemicals (Anaheim, CA, US). ABT-199 (Venetoclax/Venclexta) was manufactured by BioVision (Milpitas, CA, US), ABT-263 (Navitoclax) by Selleck Chemicals (Houston, TX, US), WEHI-539 hydrochloride was purchased from Hycultec (Beutelsbach, DE), S63845 from ApexBio (Houston, TX, US). Recombinant His^6^-tagged GFP-Annexin V was purified as previously described^49,50^. LY-294002 and rapamycin were from Enzo Life Science (Lausen, CH).

### In vitro differentiation of mouse basophils

Conditional Hoxb8-immortalized basophil committed myeloid progenitors, termed IL-3^cond^Hoxb8, were generated from bone marrow of WT mice and differentiated in vitro into mature basophils as previously described^20,21^, and are referred to as in vitro-differentiated mouse basophils throughout this manuscript. Cells were cultured in RPMI-1640 AQmedia supplemented with 10% FCS, 100 U/ml penicillin, 100 µg/ml streptomycin, 10% WEHI-3B cell-conditioned medium as a source of mouse IL-3 (ca 200 pg/ml final concentration) and 0.1 μM 4-OHT. Upon removal of 4-OHT, mature basophils were obtained within 6 days. To confirm basophil differentiation, cells were stained for the surface marker profile CD-117/c-kit^-^FcεRI^+^, using the following antibodies (all from BioLegend, San Diego CA, US): hamster anti-FcεRI (clone MAR-1) and rat anti-CD117 (c-kit, clone 2B8) and measured by flow cytometry (FACS Verse or FACS Lyric, BD BioScience)^20,21^. Data were analyzed by FlowJo V.10.1.

### Cell death measurement by flow cytometric analysis

In vitro-differentiated basophils were thoroughly washed with PBS prior to incubation with the indicated stimuli, in the presence or absence of high concentrations of recombinant mouse IL-3 (10 ng/ml) or recombinant mouse IL-4 (20 ng/ml) with or without rat anti-IL-4 (clone 11B11, BioLegend) or rat anti-IL3 (clone MP2-8F8, BioLegend) neutralizing antibodies. Basophils were stained with GFP-annexin V diluted in FACS buffer (150 mM NaCl, 4 mM KCl, 2.5 mM CaCl_2_, 1 mM MgSO_4_, 15 mM HEPES pH 7.2, 2 % FCS and 10 mM NaN_3_) for 20 min on ice in the dark. Following one washing step with FACS buffer, cells were resuspended in FACS buffer containing 2 μg/ml of PI and subsequently measured by flow cytometry (FACS Verse or FACS Lyric, BD Biosciences, San Jose, CA, US). Data were analyzed using FlowJo version 10.1., where (excluding debris and doublets) GFP-annexin V and PI double-negative cells were considered viable and displayed as percentage of overall survival at indicated time points.

### Flow cytometric analysis of surface receptor expressions

After indicated time points, basophils were collected in FACS tubes and stained on ice in the dark for 30 min: hamster anti-FcεRI (clone MAR-1) and/or IL-3Rα/CD123 (clone 5B11), anti-CD45.2 (clone 104), or IgG2aκ isotype (clone RTK2758) (BioLegend) were diluted in antibody blocking buffer containing of 3% normal rat serum (Invitrogen/ Thermo Fisher Scientific, Waltham, MA, US) and 20% 2.4G2 hybridoma conditioned medium as source of anti-CD16/CD32 FcRII/III blocking antibodies in FACS buffer. Cells were washed once prior to flow cytometric analysis using FACS Verse or FACS Lyric. Gate was set on viable single cells to analyze the variation of the geometric mean upon the indicated stimuli. All time points were set into relation of the expression level at time point zero, corresponding to 100%. Data were analyzed using FlowJo version 10.1.

### SDS-PAGE gel electrophoresis and quantitative immunoblotting

After washing with PBS, 3 × 10^6^ cells were directly lysed in pre-heated H8 buffer (20 mM Tris/HCl pH 7.5, 2 mM EGTA, 2 mM EDTA, 1% SDS, supplemented with 50 mM DTT) and boiled and homogenized at 95 °C in the presence of 4× Lämmli buffer. Proteins were separated on 12.5% or 15% denaturing SDS-PAGE gels and transferred to polyvinilydene difluoride (PVDF) membrane (Immobilon-FL, 0.45 μM, Merck Millipore, Zug, CH). After blocking, the membranes were probed overnight with the following primary antibodies: mouse anti-BCL-2 (clone 10C4, BioLegend); rat anti-BIM (clone 3C5) and rat anti-MCL-1 (clone 14C11), kind gifts from D. Huang (Parkville, Australia); mouse anti-GSK-3α/β (clone 0011-A/ 1H8) form Santa Cruz Biotechnology (Dellas, TX, US); rabbit anti-phospho-AKT (Ser473) (clone D9E) and mouse anti-AKT (clone 40D4) from Cell Signaling Technology (Danvers, MA, US); mouse anti-tubulin (clone B-5-1-2) from Sigma; mouse anti-GAPDH from Merck Millipore (Zug, CH); mouse anti-actin from BD Biosciences (San Jose, CA, US). For all immunoblots with total lysates, near-infrared fluorophore-conjugated secondary antibodies (LI-COR Biosciences, Bad Homburg, DE) were used. All immunoblots were analyzed and quantified with the Odyssey^®^ Fc Dual-Mode Imaging System using the ImageStudio software 3.1.4 (LI-COR).

### RT^2^ profiler PCR array

5 × 10^6^ basophils were incubated for 3 h in presence or absence of 20 ng/ml IL-4, collected, washed twice in PBS, and snap-frozen until further procession. Messenger RNA was isolated using the SV Total RNA Isolation System from Promega (Fitchburg, WI, US). RNA was reverse transcribed using oligo d(T) primers and M-MLV reverse transcriptase (Promega) according to the manufacturer’s instructions. RT^2^ Profiler PCR array of mouse genes related with apoptosis (PAMM-012Z) was purchased from QIAGEN (Venlo, NL). Using 5xHOT FIREPol EvaGreen qPCR master Mix Plus (Solis BioDyne, Tartu, Estonia), array was run in CFX Connect cycler with the following program: 15 min at 95 °C, 40 cycles with 15 s at 95 °C, 1 min at 60 °C followed by 1 min at 72 °C. Data analysis was performed using suppliers SA Biosciences PCR array data analysis software (http://www.sabiosciences.com/rt_pcr_product/HTML/PAMM-012Z.html) and displayed in Prism 6 generated graph.

### AKT/PKB phospho antibody array

Lysates from untreated and IL-4 exposed basophils were subjected to the AKT phospho-array (PARB216) from Full Moon BioSystems, Inc. (Sunnyvale, CA, US) according to the manufacturer’s protocol. Final analysis including slide scanning and data evaluation was performed by Full Moon BioSystems. The mean signal intensity of each spot on the array was extracted, determining the average signal intensity of all six replicated spots. The resulting average signal was than normalized to the average signal background of each slide before the phospho side-specific antibody signal was compared to the site-specific antibody. Subsequently, the data obtained were compared between IL-4 and untreated basophils and displayed using Prism 6 software (GraphPad, La Jolla, CA, USA). Complete data is shown in Supplementary Table S1, showing increase in protein phosphorylation in red, decrease in green.

### Statistical analysis

Dose response analysis of IL-4 and its effect on forward scatter (FSC)/side scatter (SSC) was analyzed by two-way analysis of variance followed by post hoc multiple comparison correction with the method of either Dunnett for the dose response or Turkey for FSC/SSC measurements. Statistical analysis of quantitative western blot data and viability kinetics were performed using multiple *t* test determining significance through Holm-Sidak correction, with *α* = 0.05. All values are represented as means ± SD. All statistical analysis was performed using Prism 6 software (GraphPad, La Jolla, CA, USA).

## Electronic supplementary material

Supplemental Figures and Tables
