# Supplementary material for: IL-4 enhances survival of in vitro-differentiated mouse basophils through transcription-independent signaling downstream of PI3K
Source: Cell Death Dis. 2018 Jun 18;9(7):713. doi: 10.1038/s41419-018-0754-z (PMC6006176; doi:10.1038/s41419-018-0754-z)
Supplement: Supplementary file 1 — Supplemental Figures and Tables [file 41419_2018_754_MOESM1_ESM.pdf]

Reinhart R et al., IL-4 enhances mouse basophil survival through transcription-independent signaling downstream of PI3K

Supplementary Figures and Tables

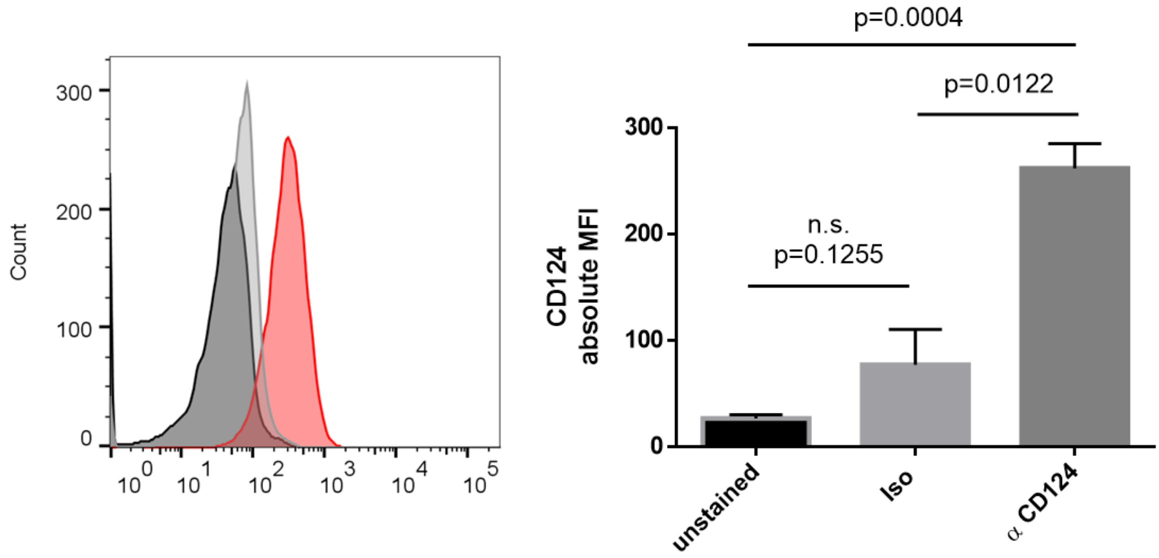

Supplementary Figure S1: IL-4 receptor expression on *in vitro* differentiated basophils. (a) Representative histogram of a flow cytometric staining of CD124, the alpha subunit of the IL-4 receptor (red), compared to unstained (black) and its corresponding isotype control (gray). Gated on single and viable cell events. (b) Mean fluorescent intensity of the geometric means of CD124 expression on *in vitro* differentiated basophils. Statistical analysis was performed using one-way ANOVA with subsequent Turkey's multiple comparison correction and displayed as means ± SD; n = 4.

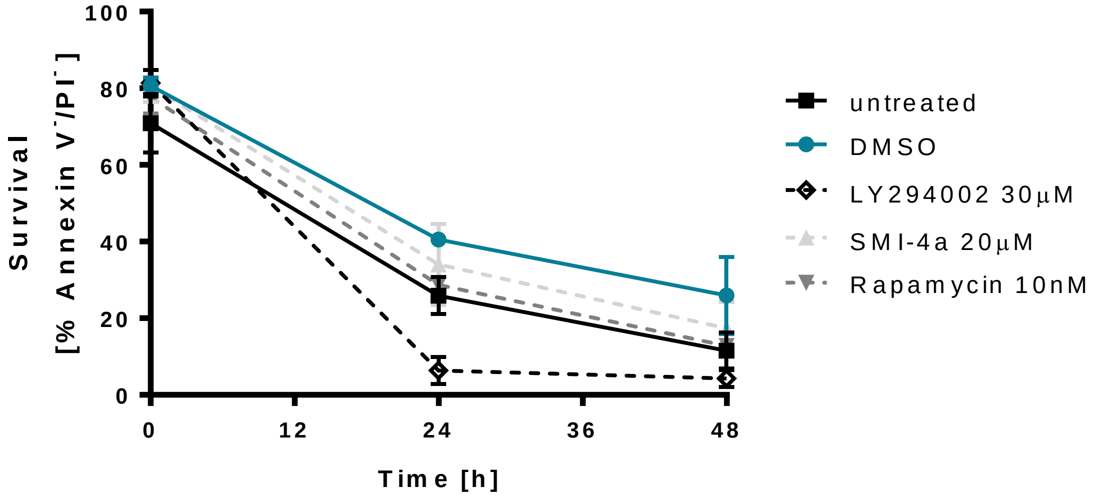

Supplementary Figure S2: DMSO does not affect the survival of *in vitro* differentiated basophils. Compared to the different kinase inhibitors as displayed within the main Figure 2, the vehicle control (DMSO) did not induce cell death, supporting the pro-survival role of PI3K in *in vitro* differentiated basophils. Means ± SD; n ≥ 4.

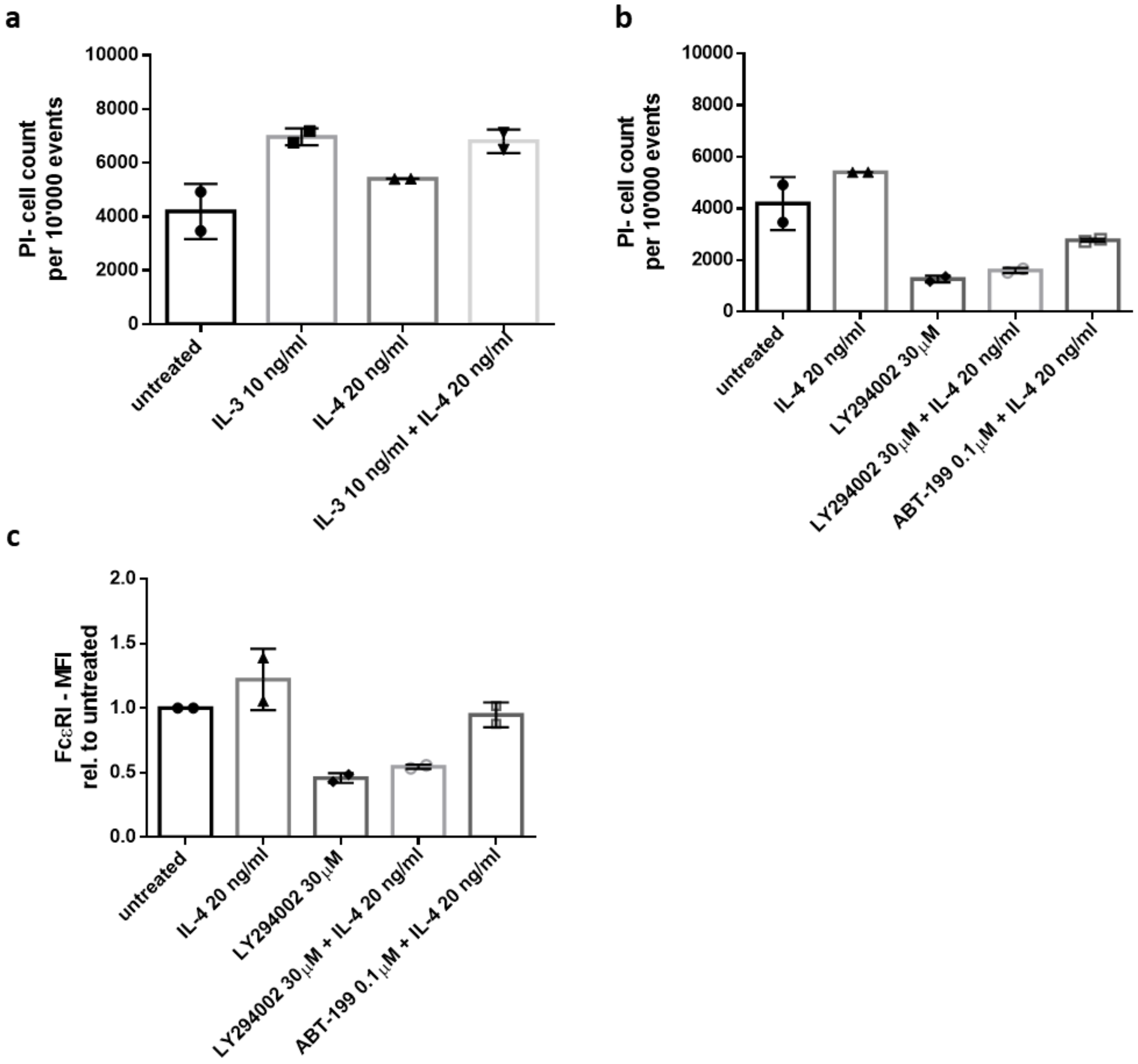

Supplementary Figure S3: PI3K contributes to IL-4-mediated survival of bone marrow derived primary mouse basophils. Primary mouse basophils (c-kit$^-$CD49b$^+$FcεRI$^+$/IgE$^+$) isolated from bone marrow of C57BL/6J WT mice were incubated for 36 hours with either 20 ng/ml IL-4, 10 ng/ml IL-3, IL-3 and IL-4 or 30 µM LY263845 with or without IL-4, or IL-4 in combination with 0.1 µM ABT-199. (a-b) Cell count of viable bone marrow derived basophils assessed by PI exclusion using flow cytometry, normalized to 10'000 measured events. (a) Cytokine mediated basophil survival upon IL-3, IL-4 or IL-3+IL-4 administration. (b) PI3K is critical for survival, as the administration of its inhibitor LY294002, with or without IL-4, decreases basophil survival. The importance of BCL-2 for primary mouse basophil survival could be confirmed using ABT-199 at a conc. of 0.1 µM. (c) Mean fluorescence intensity of FcεRI surface expression on bone marrow derived basophils, relative to untreated. Change in FcεRI surface expression upon IL-4 (20 ng/ml) and PI3K (LY294002 30 µM) or BCL-2 inhibitor (ABT-199 0.1 µM). All histograms are displaying means ± SD from two independent basophil isolations.

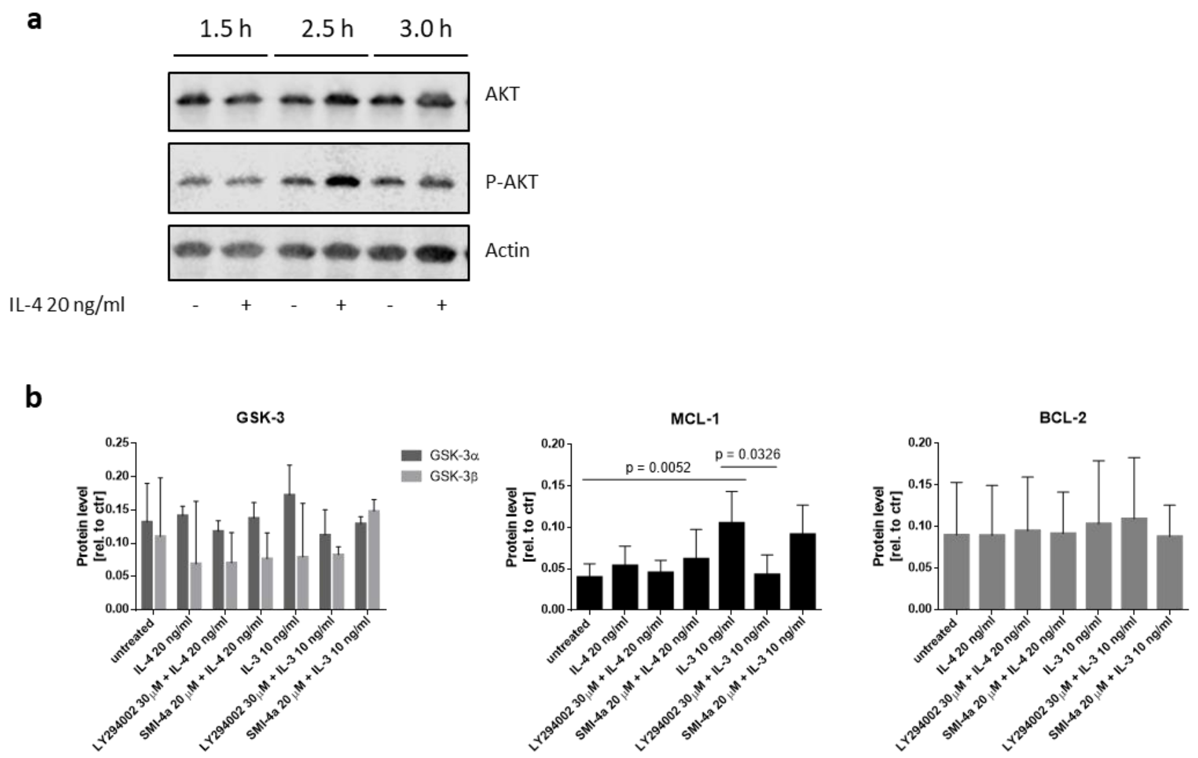

Supplementary Figure S4: Protein levels of AKT related proteins. (a) Western blot showing protein levels of *in vitro* differentiated mouse basophils after indicated time points upon treatment with and without IL-4 (20 ng/ml), depicted as a representative western blot using near-infrared fluorescence (n ≥ 2). (b) Quantitative analysis of protein expression after western blot measurements shown in Figure 3b (n ≥ 3, ± SD), significant differences are calculated by multiple t test and Holm-Sidak multiple comparison correction.

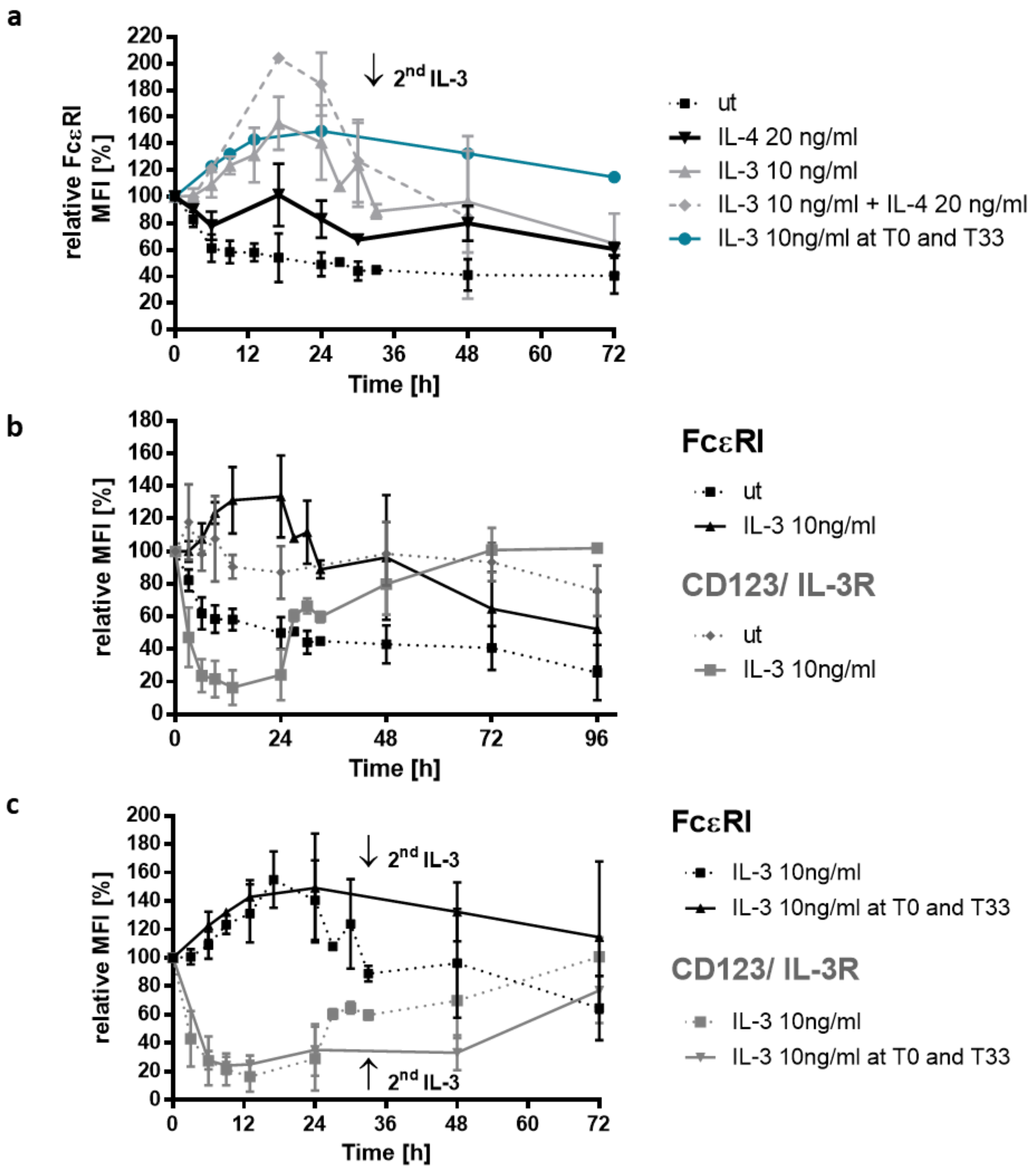

Supplementary Figure S5: Surface receptor FcεRI and IL-3R expression upon cytokine administration. (a-c) Flow cytometric quantification of (a) FcεRI expression dynamics upon cytokine administration, normalized to timepoint 0 h (n ≥ 2, ± SD). (b) Surface receptor expression of FcεRI (black) and IL-3R (grey) over time, with or without cytokine IL-3 (10 ng/ml) administration relative to timepoint 0 h (n ≥ 3, ± SD). (c) Reinforced effect of cytokine mediated receptor expression regulation of FcεRI (black) and IL-3R (grey) upon re-administration of IL-3 (10 ng/ml) after 33 h (n ≥ 2, ± SD).

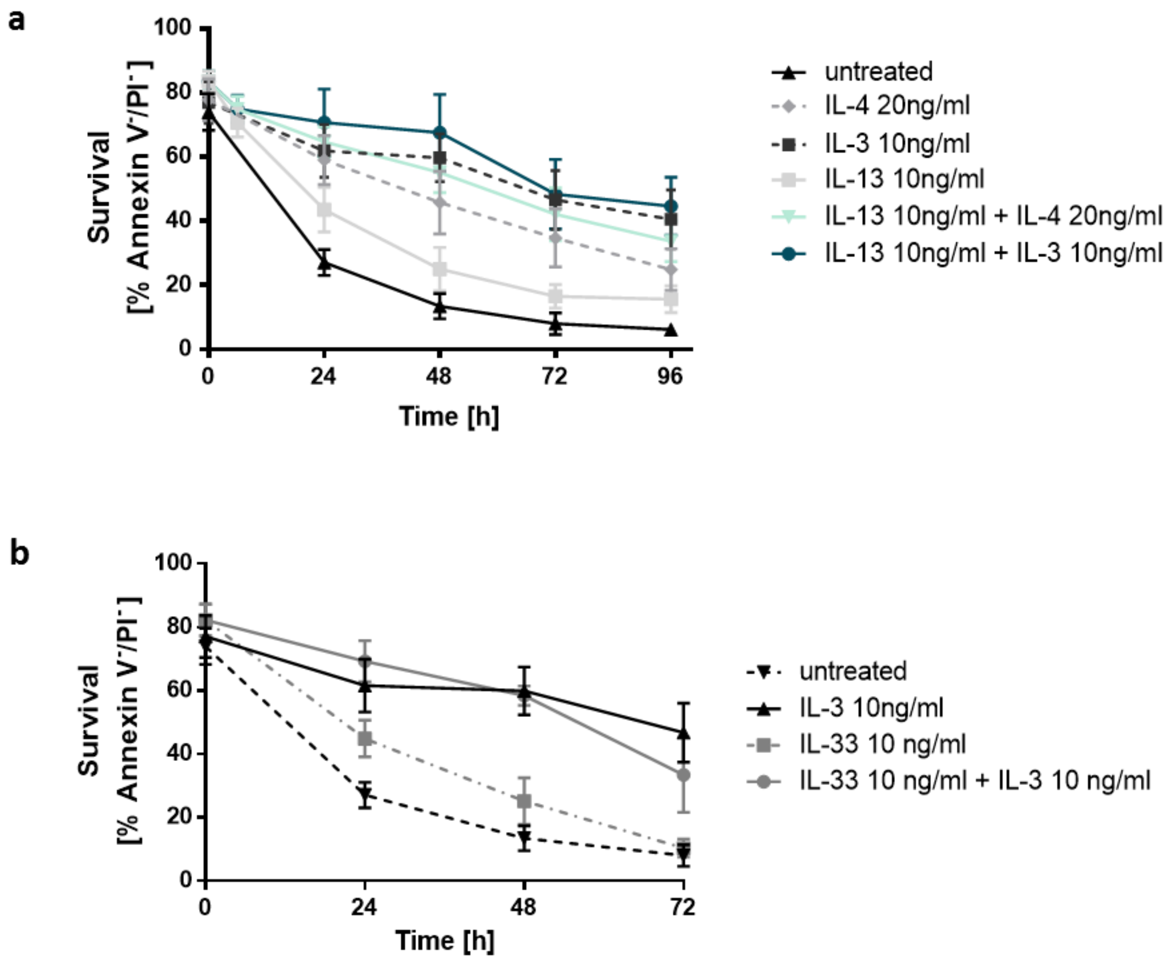

Supplementary Figure S6: Survival regulation of basophils by various cytokines. Flow cytometric measurements of basophil viability over indicated time points by Annexin V/PI exclusion (n ≥ 2, ± SD). (a) Combinational and single treatments of *in vitro* differentiated basophils with IL-13 (10 ng/ml) with and without IL-4 (20 ng/ml) or IL-3 (10 ng/ml) co-incubation. (b) Effect of IL-33 (10 ng/ml) on basophil viability over time with and without IL-3.

14-3-3 theta/tau (Phospho-Ser232)	2.48
14-3-3 zeta (Phospho-Ser58)	0.96
14-3-3 zeta/delta (Phospho-Thr232)	1.76
6-phosphofructo-2-kinase/fructose-2,6-biphosphatase 2 (PFKFB2) (Phospho-Ser483)	2.10
AKT (Phospho-Thr308)	0.93
AKT (Phospho-Tyr326)	1.11
AKT (Phospho-Ser473)	0.97
AKT1 (Phospho-Ser124)	0.92
AKT1 (Phospho-Ser246)	2.12
AKT1 (Phospho-Thr450)	0.98
AKT1 (Phospho-Tyr474)	1.78
AKT1 (Phospho-Thr72)	2.54
AKT1S1 (Phospho-Thr246)	0.80
AKT2 (Phospho-Ser474)	0.66
BAD (Phospho-Ser112)	1.21
BAD (Phospho-Ser134)	1.11
BAD (Phospho-Ser136)	1.38
BAD (Phospho-Ser155)	1.59
BAD (Phospho-Ser91/128)	1.04
BCL-2 (Phospho-Thr56)	1.07
BCL-2 (Phospho-Thr69)	2.07
BCL-2 (Phospho-Ser70)	1.03
BIM (Phospho-Ser69/65)	1.73
Cyclin D1 (Phospho-Thr286)	1.13
eNOS (Phospho-Ser1177)	1.15
eNOS (Phospho-Thr495)	1.28
eNOS (Phospho-Ser615)	1.05
FAK (Phospho-Tyr397)	1.19
FAK (Phospho-Tyr407)	0.82
FAK (Phospho-Tyr576)	0.81
FAK (Phospho-Tyr861)	1.49
FAK (Phospho-Ser910)	0.90
FAK (Phospho-Tyr925)	0.77
FKHR (Phospho-Ser256)	0.71
FKHR (Phospho-Ser319)	0.59
FOXO1/3/4-PAN (Phospho-Thr24/32)	1.07
FOXO1A (Phospho-Ser329)	0.97
Gab1 (Phospho-Tyr627)	1.98
Gab1 (Phospho-Tyr659)	1.88
Gab2 (Phospho-Tyr643)	0.86
GABA-RB (Phospho-Ser434)	0.91
GSK3a-b (Phospho-Tyr216/279)	2.25
GSK3α (Phospho-Ser21)	0.96
GSK3β (Phospho-Ser9)	0.92
IKK α (Phospho-Thr23)	0.65
IKKa/b (Phospho-Ser180/181)	0.84
IRS-1 (Phospho-Ser307)	1.41
IRS-1 (Phospho-Ser312)	0.58
IRS-1 (Phospho-Ser323)	1.11
IRS-1 (Phospho-Ser636)	1.39
IRS-1 (Phospho-Ser639)	1.26
IRS-1 (Phospho-Ser794)	2.32
JAK1 (Phospho-Tyr1022)	0.76
LYN (Phospho-Tyr507)	0.75
MDM2 (Phospho-Ser166)	0.70
mTOR (Phospho-Thr2446)	0.91
mTOR (Phospho-Ser2448)	1.44
mTOR (Phospho-Ser2481)	2.38
p21Cip1 (Phospho-Thr145)	1.15
p27Kip1 (Phospho-Ser10)	1.05
p27Kip1 (Phospho-Thr187)	0.99
p53 (Phospho-Ser15)	0.79
p53 (Phospho-Thr18)	0.79
p53 (Phospho-Ser20)	0.83
p53 (Phospho-Ser315)	0.84
p53 (Phospho-Ser33)	0.73
p53 (Phospho-Ser37)	0.71
p53 (Phospho-Ser378)	0.78
p53 (Phospho-Ser392)	1.06
p53 (Phospho-Ser46)	0.71
p53 (Phospho-Ser6)	0.84
p53 (Phospho-Ser9)	0.84
p70S6K (Phospho-Thr229)	0.97
p70S6K (Phospho-Ser371)	1.05
p70S6K (Phospho-Ser411)	1.42
p70S6K (Phospho-Ser418)	1.04
p70S6K (Phospho-Thr421)	2.10
p70S6K (Phospho-Ser424)	1.49
p70S6K-beta (Phospho-Ser423)	0.81
Paxillin (Phospho-Tyr118)	0.82
Paxillin (Phospho-Tyr31)	1.09
PDK1 (Phospho-Ser241)	0.99
PI3-kinase p85-subunit alpha/gamma (Phospho-Tyr467/Tyr199)	0.93
PP2A-a (Phospho-Tyr307)	0.65
PTEN (Phospho-Ser370)	0.97
PTEN (Phospho-Ser380)	1.10
PTEN (Phospho-Ser380/Thr382/Thr383)	1.01
S6 Ribosomal Protein (Phospho-Ser235)	1.36
SYK (Phospho-Tyr348)	2.36
SYK (Phospho-Tyr525)	1.14
SYN1-Synapsin 1 (Phospho-Ser62)	0.93
Tuberin/TSC2 (Phospho-Thr1462)	1.77
Tuberin/TSC2 (Phospho-Ser939)	1.04
XIAP (Phospho-Ser87)	0.79

Fold change in phosphorylation upon IL-4

0.5 0 2

Supplementary Table S1: Data of protein phospho-array. Protein phospho-arrays were analyzed by Full Moon array analysis service. Data represent average signal intensity of the mean intensity of six replicates, which were normalized to the average signal intensity of each slide. Color code represents the increase (red) and decrease (green) of the site-specific phosphorylation comparing IL-4 treated basophils over untreated.

		P-Value			
		**24 h**	**48 h**	**72 h**	**96 h**
**Survival**	**ut vs. LY294002**	< 0.0001	0.0026	0.0296	0.6009
	**IL-4 vs. LY294002 + IL-4**	< 0.0001	< 0.0001	< 0.0001	< 0.0001
	**IL-3 vs. LY294002 + IL-3**	0.0002	0.0015	0.0142	0.6344
	**ut vs. Rapamycin 20µM**	0.7779	0.6266	0.3290	0.1426
	**ut vs. Rapamycin 30µM**	0.7518	0.8945	0.7322	0.9147
	**IL-4 vs. Rapamycin 20µM + IL-4**	0.1729	0.3062	0.2440	0.6301
	**IL-4 vs. Rapamycin 30µM + IL-4**	0.6096	0.4085	0.1149	0.3718
	**ut vs. SMI-4a**	0.0959	0.3417	0.2222	0.4953
	**IL-4 vs. SMI-4a + IL-4**	0.0083	0.0172	0.0089	0.0058
	**IL-3 vs. SMI-4a + IL-3**	0.3986	0.3564	0.7758	0.8503

Supplementary Table S2: Statistical analysis of Figure 2 comparing potency in cell death induction over time upon protein inhibitor administration. Data analyzed by GraphPad Prism using multiple t test assuming no consistent SD followed by post hoc Holm-Sidak correction with α = 5.0 %. Comparisons are considered significant different as soon as P-values are ≤ 0.05. Non-significant comparisons at distinct time points are highlighted by a grey background.

		P-Value		
		**24 h**	**48 h**	**72 h**
**Survival**	**IL-4 vs. IL-4 + ABT-199 0.1 µM**	< 0.0001	< 0.0001	< 0.0001
	**IL-4 vs. IL-4 + ABT-199 0.01 µM**	0.00014	0.00228	0.00319
	**IL-4 vs. IL-4 + ABT-263 1 µM**	< 0.0001	< 0.0001	< 0.0001
	**IL-4 vs. IL-4 + ABT-263 0.1 µM**	0.00696	0.07392	0.04937
	**IL-4 vs. IL-4 + WEHI-539 1 µM**	< 0.0001	< 0.0001	< 0.0001
	**IL-4 vs. IL-4 + WEHI-539 0.1 µM**	0.00723	0.03159	0.01846
	**IL-4 vs. IL-4 + S63845 10 µM**	< 0.0001	0.00012	0.00061
	**IL-4 vs. IL-4 + S63845 1 µM**	< 0.0001	0.00384	0.00488

Supplementary Table S3: Statistical analysis of Figure 3 cell death induction upon BH3 mimetic treatment over time. Data analyzed by GraphPad Prism using multiple t test assuming no consistent SD followed by post hoc Holm-Sidak correction with α = 5.0 %. If P-values are ≤ 0.05, the difference between the groups are considered significant different. Non-significant comparisons at distinct time points are highlighted by a grey background.

	ut vs IL-4		ut vs IL-3		ut vs IL-3 + IL-4		IL-4 vs IL-3		IL-4 vs IL-3 + IL-4		IL-3 vs IL-4 + IL-3		IL-3 vs LY294002 + IL-3		IL-4 vs LY294002 + IL-4		IL-3 + IL-4 vs LY294002 + IL-3 + IL-4	
Time [h]	*Significance*	*P value*	*Significance*	*P value*	*Significance*	*P value*	*Significance*	*P value*	*Significance*	*P value*	*Significance*	*P value*	*Significance*	*P value*	*Significance*	*P value*	*Significance*	*P value*
3	N.S.	0.05657	N.S.	0.17871	****	< 0.0001	N.S.	0.87042	****	< 0.0001	****	< 0.0001	***	0.00047	****	< 0.0001	****	< 0.0001
6	N.S.	0.25899	N.S.	0.31213	****	< 0.0001	N.S.	0.09167	****	< 0.0001	****	< 0.0001	**	0.00375	****	< 0.0001	****	< 0.0001
24	**	0.00275	****	< 0.0001	****	< 0.0001	***	0.00027	****	< 0.0001	****	< 0.0001	****	< 0.0001	****	< 0.0001	****	< 0.0001
30	**	0.00313	****	< 0.0001	****	< 0.0001	***	0.00055	**	0.00394	****	< 0.0001	****	< 0.0001	***	0.00068	***	0.00022
48	*	0.04563	**	0.00138	****	< 0.0001	**	0.00193	N.S.	0.09419	**	0.00298	****	< 0.0001	N.S.	0.16274	*	0.01195

Supplementary Table S4: Statistical analysis of Figure 4 c + d comparing expression levels of FcεRI surface receptor on *in vitro* differentiated basophils. Data analyzed by GraphPad Prism using multiple t test assuming no consistent SD followed by post hoc Holm-Sidak correction with α = 5.0 %. Difference between different stimuli is considered significant if * P ≤ 0.05, ** P ≤ 0.01, *** P ≤ 0.001, **** P ≤ 0.0001; N.S. not significant highlighted by grey background.
